# A Hand-book of Obstetric Surgery

**Published:** 1874-10

**Authors:** 


### Books Reviewed.

*A Complete Hand-Book of Obstetric Surgery; Or short Rules of Practice in every Emergency from the Simplest to the most Formidable Operations connected with the Science of Obstetricy.* By CHARLES CLAY, M. D., etc. Philadelphia: Lindsay & Blakiston, 1874.

Dr. Clay's Hand-book has met, in England with merited favor, and has there reached its third edition. The author has endeavored to make this a hand-book or remembrancer of those points in the Obstetric Art upon which practitioners should be informed, but upon which slight additional intelligence may be required at a moments notice, when the time could not be spared to search through the more comprehensive treatises. It will be found that Dr. Clay has collected within the narrow limits of this work all the points liable to tax the art of the obstetrician in an emergency; and to those who desire to satisfy their minds at a moments notice this little work will prove invaluable. The directions are clear, but the operative procedures are not so minutely described that pages of the book will not have to be perused to get a desired point. The author has written with the idea that his reader has a general knowledge of the whole subject, and that his pages will only be sought as a ready means of gaining information upon some doubtful or forgotten point.

